# Characterization of the nasal and oral microbiota of detection dogs

**DOI:** 10.1371/journal.pone.0184899

**Published:** 2017-09-21

**Authors:** Anitha Isaiah, Aline Rodrigues Hoffmann, Russ Kelley, Paul Mundell, Jörg M. Steiner, Jan S. Suchodolski

**Affiliations:** 1 Gastrointestinal Laboratory, Department of Small Animal Clinical Sciences, College of Veterinary Medicine and Biomedical Sciences, Texas A&M University, College Station, Texas, United States of America; 2 Dermatopathology Specialty Service, Department of Veterinary Pathobiology, College of Veterinary Medicine and Biomedical Sciences, Texas A&M University, College Station, Texas, United States of America; 3 Private consultant, Waynesville, Ohio, United States of America; 4 Canine Companions for Independence, Santa Rosa, California, United States of America; University of Oklahoma, UNITED STATES

## Abstract

Little is known about physiological factors that affect the sense of olfaction in dogs. The objectives of this study were to describe the canine nasal and oral microbiota in detection dogs. We sought to determine the bacterial composition of the nasal and oral microbiota of a diverse population of detection canines. Nasal and oral swabs were collected from healthy dogs (n = 81) from four locations—Alabama, Georgia, California, and Texas. Nasal and oral swabs were also collected from a second cohort of detection canines belonging to three different detection job categories: explosive detection dogs (SP-E; n = 22), patrol and narcotics detection dogs (P-NDD; n = 15), and vapor wake dogs (VWD-E; n = 9). To understand if the nasal and oral microbiota of detection canines were variable, sample collection was repeated after 7 weeks in a subset of dogs. DNA was extracted from the swabs and used for 454-pyrosequencing of the16S rRNA genes. Nasal samples had a significantly lower diversity than oral samples (*P*<0.01). Actinobacteria and Proteobacteria were higher in nasal samples, while Bacteroidetes, Firmicutes, Fusobacteria, and Tenericutes were higher in oral samples. Bacterial diversity was not significantly different based on the detection job. No significant difference in beta diversity was observed in the nasal samples based on the detection job. In oral samples, however, ANOSIM suggested a significant difference in bacterial communities based on job category albeit with a small effect size (R = 0.1079, *P* = 0.02). Analysis of the composition of bacterial communities using LEfSe showed that within the nasal samples, *Cardiobacterium* and *Riemerella* were higher in VWD-E dogs, and *Sphingobacterium* was higher in the P-NDD group. In the oral samples *Enterococcus* and *Capnocytophaga* were higher in the P-NDD group. *Gemella* and *Aggregatibacter* were higher in S-PE, and *Pigmentiphaga*, *Chryseobacterium*, *Parabacteroides* amongst others were higher within the VWD-E group. Our initial data also shows that there is a temporal variation in alpha diversity in nasal samples in detection canines.

## Introduction

It is estimated that the olfactory perception of dogs is about 10,000 times more sensitive than that of man [1]. This remarkable ability of dogs is beneficial to humans in a variety of ways, as dogs can be trained and utilized for various detection jobs like detection of explosives and narcotics, search and rescue, as well as other duties that require an extremely developed olfactory system [2]. Over the past years, there has been a significant interest in research to improve the olfactory performance of working canines.

Studies have shown that the microbiota can have an impact on health, immune homeostasis, nutrient status [3] and the cognitive function [4] of the host. The majority of those studies have focused on the microbiota of the gastrointestinal tract; however, there is sufficient evidence suggesting that the microbiota associated with skin [5] and the oral cavity [6] also has crucial health implications on the host [5, 7, 8].

Microbes can play a role in influencing group-specific social orders mediated via olfaction. For example, in hyena populations, members of the same family clans harbor more similar microbial communities in their anal glands than members of other family clans [9]. The scent secretions from these microbial communities in the anal glands are factors that allow family members to recognize other members of their clan [9], indicating the importance of the microbiota in olfactory signaling. Studies also show that signals derived from gut microbiota can influence the olfactory receptors in tissues outside the olfactory system [10]. Recent literature shows that the manipulation of the gut microbiota in mice by changing the diet [11] or by introducing antimicrobials [12] could lead to changes in cognition, behavior, and gene expression levels in the brain. Studies in germ-free mice have shown that the microbiota can modulate the physiology of the olfactory epithelium [13]. Jenkins *et al*. reported that the oral administration of metronidazole altered the detection ability of explosive detection dogs [14]. These findings suggest that microbial communities could influence host physiology and behavior. Studies show that when compared to the gut microbiota, host skin microbiota is more dependent on geographic location [14]. A potentially unfavorable outcome, especially in the case of military working detection dogs, would be if transportation of detection dogs to different locations could alter their nasal and oral microbiota, and thereby affect their olfactory detection skills.

Previous studies have described the canine nasal [15, 16] and oral [16–19] microbiota, using culture dependent and culture independent methods. However, most of these studies were focused on pet dogs. The goals of the study were, 1) to further describe the resident nasal and oral microbiota in a larger and more diverse population of dogs from different geographic locations, and 2) to compare the nasal and oral microbiota of dogs with different detection jobs and to assess the temporal stability of nasal and oral microbiota in these dogs after 7 weeks.

## Methods

### Animal enrollment and sample collection

To describe the nasal and oral microbiota of healthy dogs from different settings, a total of 149 swabs were collected from 81 healthy dogs (cohort-1) from four locations: Alabama (military/law enforcement working dogs), Georgia (upland sporting dogs), California (service dogs) and Texas (pet dogs). From these, we collected 69 nasal and 80 oral swabs. Sterile swabs were inserted approximately half an inch in the nasal cavity and swabbed. For oral samples, buccal swabs were collected. From each site, two swabs were collected and immediately transferred to the lysis buffer from Mobio Power Soil DNA Extraction kit (MoBio Laboratories, Inc., CA). Paired samples were not collected from some of the enrolled dogs since they did not co-operate with the sample collection.

Nasal (n = 34) and oral (n = 46) samples were collected from a second cohort (cohort-2) of dogs to describe the microbiota of dogs with different detection jobs. These dogs were all from a single training facility and were on the same diet. These dogs were trained for specific detection tasks like explosives detection (SP-E), patrol and narcotics detection (P-NDD) and vapor wake detection (VWD-E). The SP-E dogs are more proficient in locating explosive devices, while the VWD-E dogs can work off the leash and can detect very dilute odors. Dogs from the P-NDD group are placed in law enforcement and are skilled in detecting narcotic substances.

To understand the temporal stability of the nasal and oral microbiota in detection dogs, nasal and oral samples were collected at a second-time point from cohort-2, approximately 7 weeks after the first collection. The metadata for all dogs enrolled in the study are listed in S1 and S2 Tables.

To control for contamination, an unused swab was also processed along with the nasal and oral swabs. The IACUC review committee at Texas A&M University (AUP 2014–0065 CA) and the Department of the Defense, Office of Naval Research (#NRD-903), approved the collection of samples.

### DNA isolation

DNA was extracted from the swabs with a MoBio Power soil DNA isolation kit (MoBio Laboratories, USA) following the manufacturer’s instructions.

### Sequencing of 16S rRNA genes

Bacterial tag-encoded FLX-Titanium amplicon pyrosequencing targeting the V4–V6 region of the 16S rRNA gene was performed at MR DNA Laboratory, Shallowater, TX, USA using the forward and reverse primers: 530F (5’-GTGCCAGCMGCNGCGG-3’) and 1100R (5’-GGGTTNCGNTCGTTG-3’). Raw sequence data was screened, trimmed, filtered, denoised and barcodes and chimera sequences were removed from the dataset using QIIME v1.8 [20] pipeline and UCHIME [21]. Operational Taxonomic Units (OTUs) were assigned based on at least 97% sequence similarity against the Greengenes 13_8 reference database [22]. For downstream analysis, sequences assigned as chloroplast, mitochondria and Unassigned were removed. Additionally, OTUs that were assigned to the phylum cyanobacteria were considered to be potential plant chloroplast contaminants and excluded from the analysis. Sequences were rarefied to an even sequencing depth of 4,600 sequences/sample in cohort-1 and 4,960 sequences/sample in cohort-2 to account for unequal sequencing depth across samples. The sequences were deposited in SRA under the accession numbers: SRP060357 and SRP072443.

### Statistical analysis

Differences in bacterial communities between nasal and oral swabs were analyzed using the phylogeny-based UniFrac distance metric and visualized with PCoA plots; rarefaction curves showing alpha diversity indices (Chao1, Shannon and Observed OTUs) were generated within QIIME [20]. ANOSIM (Analysis of Similarity) test within PRIMER 6 software package (PRIMER-E Ltd., Luton, UK) was used to analyze significant differences in microbial communities between the nasal and oral samples. ANOSIM test compares microbial community composition between samples, wherein microbial communities that are different will have an R statistic near 1 and similar microbial communities will have an R statistic nearing 0. The data were tested for normality using the Shapiro-Wilk test (JMP Pro 11, SAS software Inc.). Most of the datasets did not meet the assumptions of normality hence Mann-Whitney U test or Kruskal-Wallis test was used. Subsequently, statistical analysis was performed using Calypso [23]. The resulting *P*-values were adjusted for multiple comparisons by Benjamini & Hochberg’s False Discovery Rate (FDR), and an adjusted *P*<0.05 was considered statistically significant [24].

Linear discriminant analysis effect size (LEfSe) was used to elucidate bacterial taxa (16S rRNA genes) associated with nasal or oral samples [25]. LEfSe was used online in the Galaxy workflow framework.

## Results

### Sequence analysis

The sequences obtained from cohort-1 were rarified to an even sequencing depth of 4,600 sequences per sample to adjust for uneven sequencing depth across the samples. From dogs in cohort-1 (n = 81), 69 nasal samples and 80 oral samples were collected. Three oral samples had to be excluded from the analysis as these samples had low sequence yield and did not reach the rarefaction depth. From dogs in cohort-2 (n = 46), 81 samples were collected nasal = 34; oral = 46) and were retained for downstream analysis. An additional 82 samples from time point 2 were collected (nasal = 29; oral = 41). From these only paired samples with a matching time point 1 sample were retained. The samples were rarefied to 4,960 sequences per sample. The summary of the sequence analysis is given in Table 1.

**Table 1:** Summary of sequencing runs. Tabular data given for the number of samples sequenced, OTUs identified and high-quality sequencing reads retained after filtering.

Summary	Cohort-1		Cohort-2			
	Nasal	Oral	Time point 1		Time point 2	
			Nasal	Oral	Nasal	Oral
Number of samples	69	80	34	46	29	41
Total number of high-quality sequences after filtering	1,493,128	1,363,045	754,974	1,107,926	990,879	1,067,354
**Sequencing reads per sample**						
Minimum	4,127	1,534	5,116	7,704	5,784	2,216
Maximum	57,824	40,111	71,037	115,883	172,947	115,883
Median	18,130	15,149	18,148	15,514	17,831	15,103
Mean	21,330	16,827	22,205	24,085	28,310	23,203
Standard deviation	13,478	8,852	15,254	26,275	30,870	26,938

### Microbiota of the nasal and oral cavities

**Fig 1:**
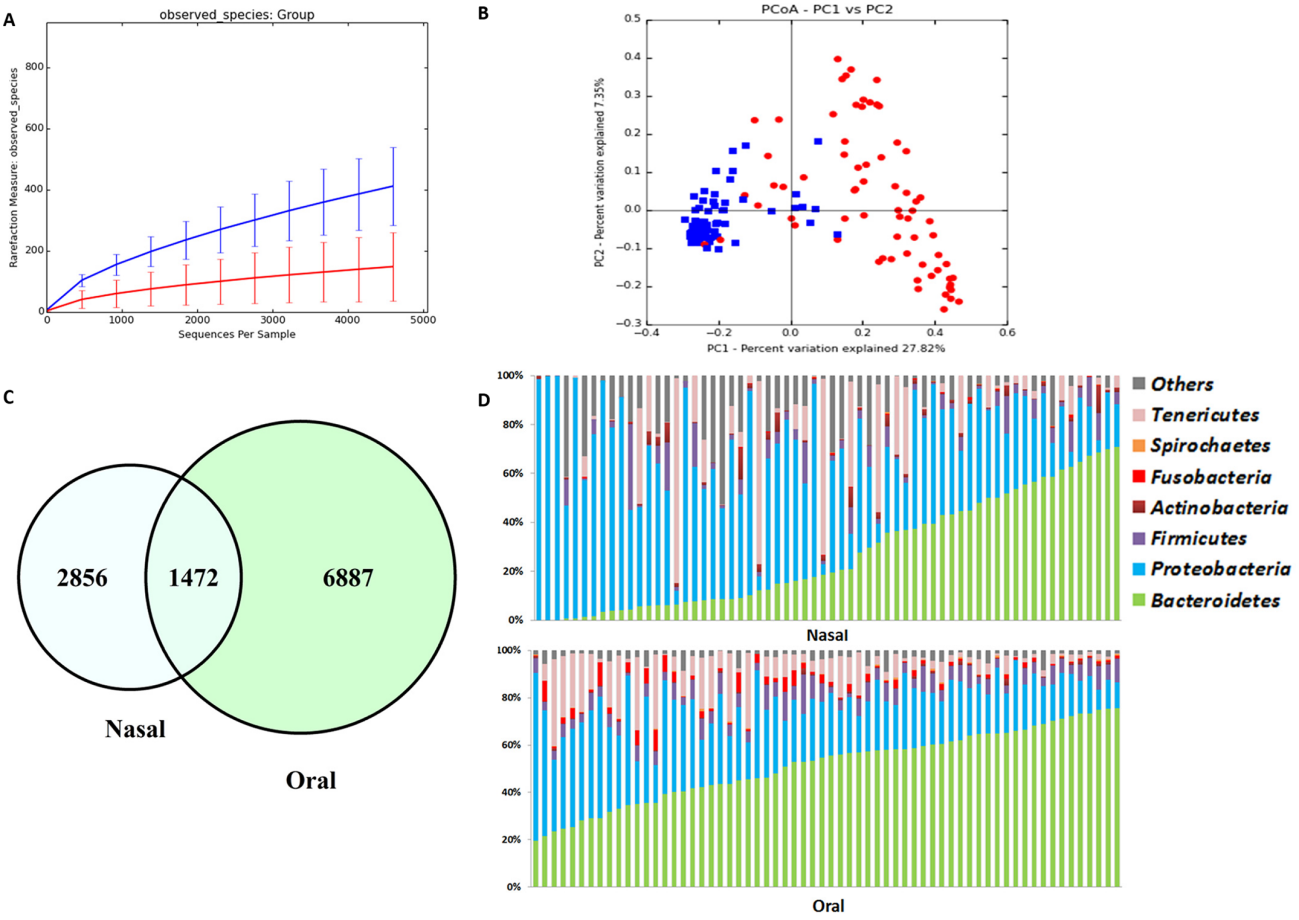
Bacterial diversity measures for all samples from cohort-1. (A) Alpha diversity: rarefaction analysis (number of observed species) of 16S rRNA gene sequences. Lines represent the mean of each group, while the error bars represent the standard deviations. (B) Beta diversity: Principal coordinate analysis (PCoA) of unweighted UniFrac distances of 16S rRNA genes. Analysis of similarity (ANOSIM) revealed clustering between oral and nasal samples (R = 0.6980; *P* = 0.01). (C) Comparison of OTUs between nasal and oral samples. A Venn diagram showing overlapping OTU’s between nasal and oral samples. (D) Bacterial phyla in nasal versus oral samples. Most common bacterial phyla identified in nasal and oral samples from healthy dogs sorted by the phylum Bacteroidetes.

Alpha diversity indices for all samples are summarized in Table 2. The rarefaction curve for observed OTUs is shown in Fig 1. Alpha diversity as described by Chao 1, Observed OTUs (species richness), and Shannon diversity index was significantly higher (*P*<0.001) in the oral samples when compared to the nasal samples.

**Table 2:** Summary of alpha diversity indices at a depth of 4,600 sequences per sample based on the site of sampling.

OTU picking method	Nasal	Oral	*P* value*
**Open reference**			
Chao1	240(82–1118)	896(359–1656)	<0.001
Observed species	102(39–603)	374(164–644)	<0.001
Shannon	2.6(0.2–6.5)	5.5(3.3–6.7)	<0.001
**Closed reference**			
Chao1	71(2–708)	193(78–330)	<0.001
Observed species	55(2–475)	148(57–252)	<0.001
Shannon	2(0.06–6.2)	4.88(2.6–6.2)	<0.001

*P values determined by Mann-Whitney U test significance level <0.05)

PCoA analysis (Fig 1, Panel B) based on unweighted UniFrac distance metric showed a distinct separation between nasal and oral samples. Bacterial community composition was significantly different as revealed by ANOSIM (R = 0.5736, *P* = 0.01). Individual bacterial groups were analyzed using a Kruskal-Wallis test (S3 Table), and several bacterial taxa were significantly different between nasal and oral samples. The similarities and differences in bacterial composition between the nasal and oral samples were visualized using a Venn diagram (Fig 1, Panel C) and showed that there were 1,472 OTUs shared between the two groups of samples. OTUs unique to the nasal and oral samples were 2,856 and 6,887 respectively. Community composition at the phylum level showed a dog-to-dog variation (Fig 1, Panel D). The predominant bacterial phyla in nasal samples were Proteobacteria followed by Bacteroidetes, whereas oral samples were mostly composed of Bacteroidetes followed by Proteobacteria. Alphaproteobacteria, Actinobacteria, and Gammaproteobacteria were significantly higher in nasal samples while Bacteroidia, Mollicutes, Betaproteobacteria, and Flavobacteriia were higher in oral samples (*P*<0.001). At the genus level, *Moraxella*, *Leucobacter*, *Helcococcus*, and *Cardiobacterium* were significantly higher in nasal samples while the genera *Porphyromonas*, *Capnocytophaga*, *Mycoplasma*, and unclassified genera in the families Neisseriaceae and Pasteurellaceae were significantly higher in oral samples (*P*<0.001).

#### Effect of geographical location on the canine nasal microbiota

**Fig 2:**
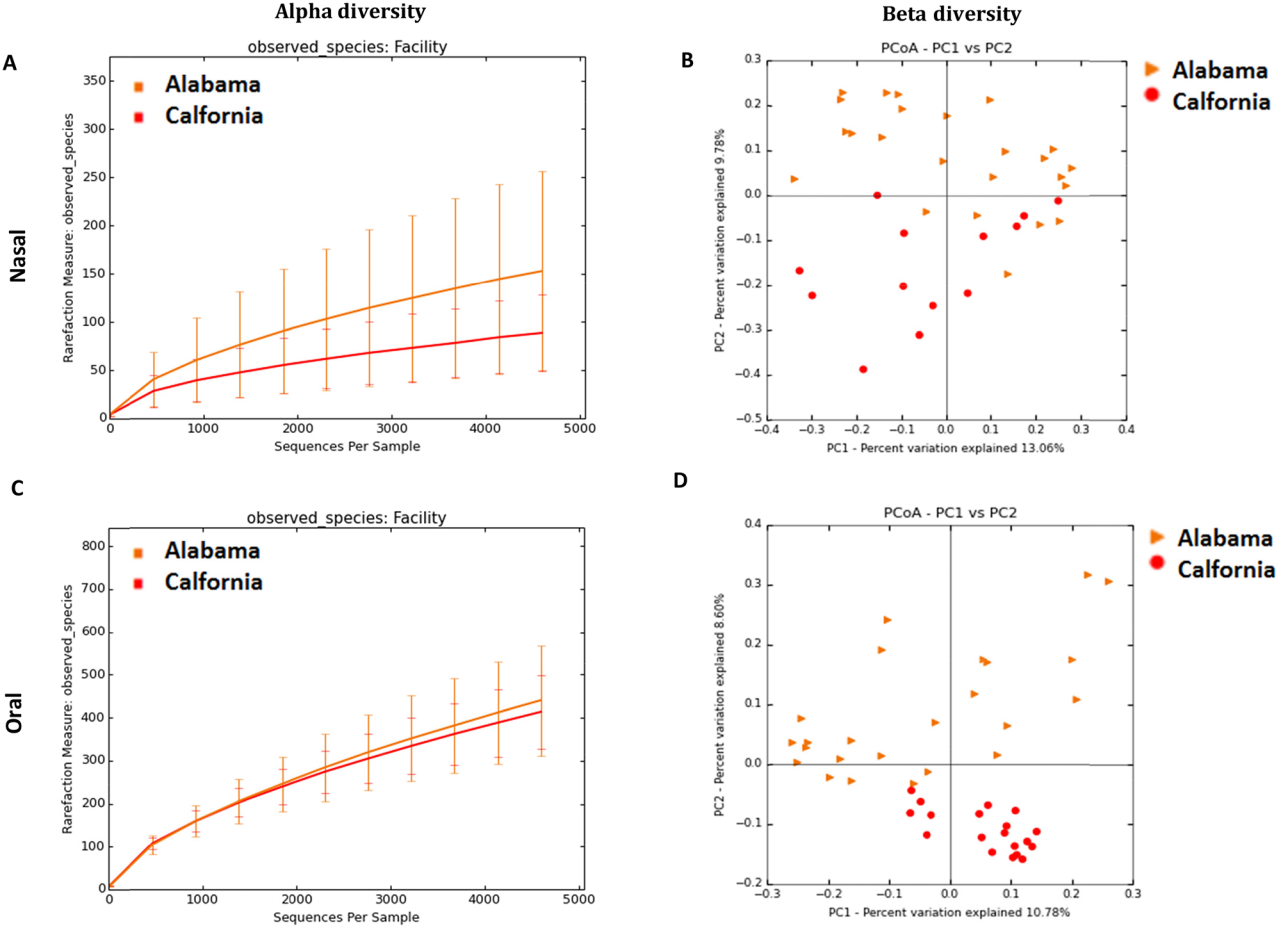
Bacterial diversity measures of samples from Labradors based on location. Alpha diversity measures: rarefaction analysis (number of observed species) of 16S rRNA gene sequences in nasal and oral samples. Lines represent the mean of each group, while the error bars represent the standard deviations. Beta diversity: Principal coordinate analysis (PCoA) of unweighted UniFrac distances of 16S rRNA genes. Analysis of similarity (ANOSIM) revealed clustering based on location for B) Nasal (R = 0.2275; *P* = 0.01) and D) Oral samples (R = 0.2008; *P* = 0.01).

When all the dogs in cohort-1 were analyzed, there was a significant difference in alpha (*P*<0.05) and beta diversity (ANOSIM; R = 0.2598; *P* = 0.01) based on location. However, to disentangle the effect of breeds vs. location, we analyzed only Labradors from two locations, Alabama and California, as these were the only two locations with a sufficient number of dogs of the same breed. The diversity indices, Observed OTUs and Chao1 (richness) were significantly lower (*P*<0.05) for the nasal samples from California when compared to Alabama. Alpha diversity measures are summarized in Table 3 and rarefaction plot for observed OTUs is shown in Fig 2 (Panel A). PCoA plot (Fig 2, Panel B) visually show that the bacterial communities cluster significantly different based on location (ANOSIM; R = 0.2275; *P* = 0.01).

**Table 3:** Summary of alpha diversity indices of samples from Labradors at a depth of 4,600 sequences per sample based on location.

	Median (Min-Max)		
**Nasal**	**Alabama (n = 24)**	**California (n = 13)**	*P* value*
Observed species	107(77–603)	74(39–159)	0.003
Shannon	2.69(1.7–6.52)	2.4(0.3–5.1)	0.309
Chao1	260(145–1089)	205(93–317)	<0.004
**Oral**	**Alabama (n = 24)**	**California (n = 19)**	***P* value**
Observed species	416(195–545)	350(290–531)	0.385
Shannon	5.6(3.3–6.6)	5.7(5.3–6.7)	0.221
Chao1	977(457–1379)	863(524–1360)	0.163

*P values determined by Kruskal Wallis test (significance level <0.05)

#### Effect of geographical location on the canine oral microbiota

When the oral samples from dogs in cohort-1 from four different locations were analyzed, Shannon diversity index, a measure of alpha diversity and beta diversity were significantly different based on location (R = 0.146; *P* = 0.01). To understand if this was a true location vs. breed effect, as described above for the nasal samples from these dogs, chose only the Labrador dogs from these two locations (Alabama and California). There was no significant difference in alpha diversity indices (Table 3) in oral samples; alpha rarefaction plot for observed OTUs is shown in Fig 2 (Panel C). PCoA plots (Fig 2, Panel D) show that the bacterial communities cluster significantly different based on location (ANOSIM; R = 0.2008; *P* = 0.01) for oral samples.

There were no significant differences based on gender in the bacterial diversity or community composition (*P*>0.05) in nasal and oral samples in the same set of Labradors within cohort-1.

### Nasal and oral microbiota of dogs with different detection jobs

To understand the nasal and oral microbiota of dogs with specific detection jobs, the following analyses were performed on samples from a second cohort (cohort-2) of dogs that lived in a single training facility and were on the same diet. As in cohort-1, the bacterial diversity in nasal samples was significantly lower (*P*<0.05) than that of oral samples (S1 Fig, Panel A). The samples also clustered based on the sampling site (nasal or oral) in cohort-2 as well (S1 Fig, Panel B). There was also an inter-dog variability in nasal and oral microbiota (S2 Fig) in the detection dogs similar as observed in dogs from cohort-1.

#### Nasal samples

**Fig 3:**
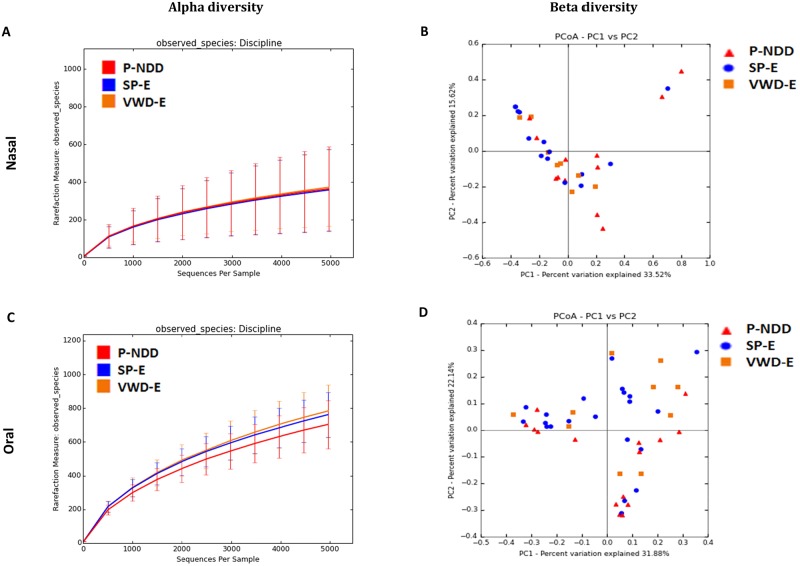
Bacterial diversity measures of samples based on job category of detection dogs in cohort 2 from nasal and oral samples. Lines represent the mean of each group, while the error bars represent the standard deviations. Beta diversity: Principal coordinate analysis (PCoA) of unweighted UniFrac distances of 16S rRNA genes. Analysis of similarity (ANOSIM) for B) nasal (R = 0.05; *P* = 0.19) and D) oral samples (R = 0.11, *P* = 0.02).

In cohort 2, there was no significant difference in alpha diversity based on detection jobs (Fig 3, Panel A). Based on PCoA plots (Fig 3, Panel B) there was no significant difference in microbial communities between the dogs belonging to the three different detection job categories based on ANOSIM test (R = 0.0469; *P* = 0.19). Based on LEfSe (Table 4), the family Cardiobacteriaceae was enriched in the VWD-E group of dogs while the family Enterococcaeceae was enriched in P-NDD group of dogs. At the genus level, the genera *Cardiobacterium*, *Riemerella* and an unclassified genus within the order Cardiobacteriales was more abundant in the P-NDD dogs while *Sphingobacterium* and an unclassified genus within Enterobacteriaceae were associated with the VWD-E group of dogs. However, univariate statistics did not show a significant difference in bacterial taxa (S4 Table).

**Table 4:** Linear discriminant analysis of bacterial genera and their associations with detection jobs. Only LDA scores of >3.0 are shown.

	LDA	Category
**Taxa**		
**Nasal**		
Unclassified_Cardiobacteriales	3.69	VWD-E
*Sphingobacterium*	3.72	P-NDD
Unclassified_Enterobacteriaceae	3.93	P-NDD
*Riemerella*	4.18	VWD-E
*Cardiobacterium*	4.67	VWD-E
**Oral**		
Unclassified_Leptotrichiaceae	2.94	VWD-E
Unclassified_Aerococcaceae	3.35	SP-E
*Aggregatibacter*	3.12	SP-E
*Enterococcus*	3.26	P-NDD
*Gemella*	3.27	S-PE
*Parabacteroides*	3.3	VWD-E
Unclassified_Clostridiales	3.42	VWD-E
*Chryseobacterium*	3.61	VWD-E
*Pigmentiphaga*	3.63	VWD-E
Unclassified_BD15	3.77	VWD-E
Unclassified_Neisseriaceae	4.18	VWD-E
*Mycoplasma*	4.21	VWD-E
Unclassified_Lachnospiraceae	4.4	P-NDD
*Conchiformibius*	4.41	VWD-E
*Capnocytophaga*	4.77	P-NDD

#### Oral samples

There was no significant difference in alpha diversity indices in oral samples based on detection jobs (Fig 3, Panel C). PCoA plots did not show a clear clustering based on the detection jobs (Fig 3, Panel D). However, based on ANOSIM test there was a significant difference in microbial communities between the dogs belonging to the three different detection job categories (R = 0.1079, *P* = 0.02). Based on LEfSe (Table 4), the genera *Capnocytophaga*, *Enterococcus*, and an unclassified genus within the family Lachnospiracea were associated with the P-NDD group of dogs while *Gemella*, *Aggregatibacter*, and an unclassified genus within Aerocoocaceae were associated with the SP-E group of dogs. *Conchiformibius*, *Pigmentiphaga*, *Chryseobacterium*, *Mycoplasma*, and *Parabacteroides* were enriched in the VWD-E dogs. However, univariate statistics did not show a significant difference in bacterial taxa (S5 Table).

#### The effect of age, breed, gender on nasal and oral microbiota in detection dogs

The effect of confounding factors such as the effect of age, breed, and gender was assessed in cohort-2, as all dogs lived in the same environment and were on the same diet. A total of 47 dogs were sampled and ranged in age from 0.5 to 6 years. The majority of the dogs belonged to the breeds Belgian Malinois (n = 14), German Shepherd (n = 10), and Labrador Retriever (n = 17). No significant differences were observed in bacterial communities in nasal samples based on age group, breed, and sex of the detection dogs. A significant difference was seen in bacterial communities in oral samples based on age group and breed (*P*<0.05) while there was no significant difference based on the sex of the detection dogs (S6 Table).

#### Temporal variability of nasal and oral microbiota of detection canines

**Fig 4:**
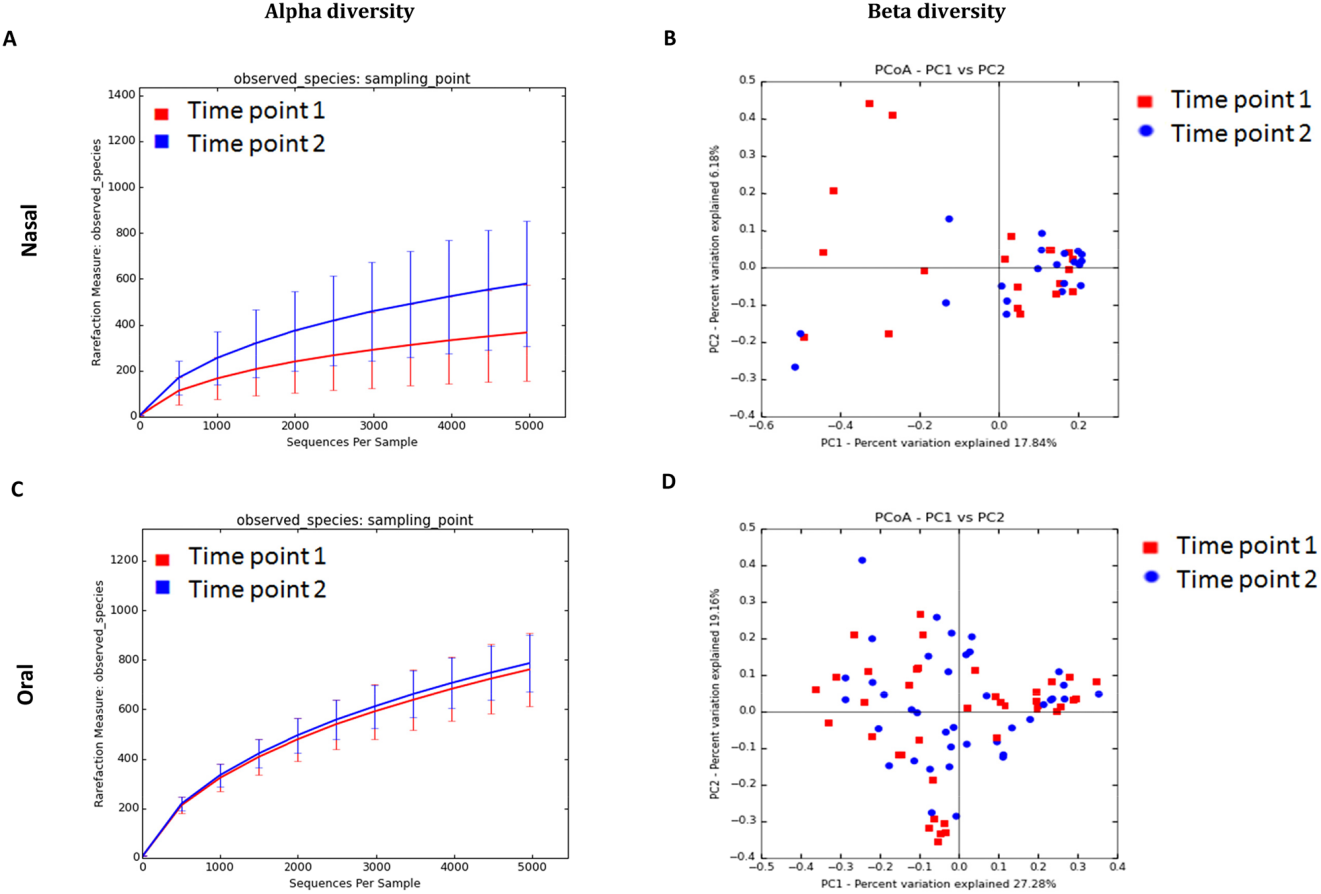
Bacterial diversity measures of samples based on sampling time point in cohort-2 from nasal and oral samples. Lines represent the mean of each group, while the error bars represent the standard deviations. Beta diversity: Principal coordinate analysis (PCoA) of unweighted UniFrac distances of 16S rRNA genes. Analysis of similarity (ANOSIM) for B) (R = 0.0272; *P* = 0.160) and D) oral samples (R = -0.0071, *P* = 0.582).

In order to understand the temporal stability of nasal and oral microbiota of detection canines, a second sample was collected from dogs in cohort-2, seven weeks after the first collection. Paired samples from both time points from 20 dogs were analyzed for nasal samples and 40 dogs for oral samples. In nasal samples, there was a significant difference in diversity based on the sampling time point (Fig 4, Panel A). There was a higher diversity in the samples when sampled at the second time point (*P*<0.01). PCoA plots do not show a clustering pattern based on sampling time point (Fig 4, Panel B), which was further confirmed with ANOSIM (R = 0.0272; *P* = 0.160). Based on LEfSe, a few bacterial genera were higher at time point 2 (Table 5). *Chryseobacterium*, *Fimbriimonas*, *Halomonas*, *Paludibacter*, and *Porphorymonas* were amongst the bacteria that were higher in the nasal samples at the second time point. However, analysis of bacterial groups using univariate testing (Wilcoxon signed rank test) did not show any significant differences in the abundances of bacterial groups between time points 1 and 2 at different taxonomic levels (data not shown).

**Table 5:** Linear discriminant analysis of bacterial genera and their associations with the sampling time. Only LDA scores of >3.0 are shown.

Taxa	LDA	Category
**Nasal**		
Unclassified_Sphingomonadaceae	3.02	Time point 2
Unclassified_F16	3.13	Time point 2
Unclassified_Peptostreptococcaceae	3.15	Time point 2
*Agrobacterium*	3.19	Time point 2
*Caulobacter*	3.22	Time point 2
Unclassified_Oxalobacteraceae	3.25	Time point 2
Unclassified_CW040	3.28	Time point 2
*Fimbriimonas*	3.29	Time point 2
Unclassified_TM71	3.31	Time point 2
Unclassified_Sphingobacteriaceae	3.33	Time point 2
Unclassified_Comamonadaceae	3.35	Time point 2
*Erwinia*	3.39	Time point 2
*Pseudomonas*	3.47	Time point 2
*Pseudoxanthomonas*	3.47	Time point 2
Unclassified_Caulobacteraceae	3.53	Time point 2
*Halomonas*	3.58	Time point 2
*Sphingomonas*	3.58	Time point 2
Unclassified_Xanthomonadaceae	3.58	Time point 2
*Chryseobacterium*	3.59	Time point 2
Unclassified_Moraxellaceae	4.16	Time point 2
*Porphyromonas*	4.56	Time point 2
**Oral**		
Unclassified_Erysipelotrichaceae	2.79	Time point 1
*Fusibacter*	2.85	Time point 2
Unclassified_CW040	3.14	Time point 2
*Tannerella*	3.15	Time point 2
*Pasteurella*	3.31	Time point 1

In oral samples (n = 80), there was no significant difference in alpha diversity based on the sampling time point (Fig 4, Panel C). As evident by the lack of clustering in the PCoA plots (Fig 4, Panel D), no significant difference in microbial communities was observed with ANOSIM (R = -0.0071, *P* = 0.582). LEfSe identified the genus *Pasteurella* to be enriched in the first sampling time point and *Fusibacter* and *Tannerella* at the second time point (Table 5). Univariate statistics (Wilcoxon signed rank test) did not show any significant differences in the abundances of bacterial groups between time points 1 and 2 (data not shown).

## Discussion

The focus of this study was to describe the canine nasal and oral microbiota in a larger population of dogs from different geographic locations. Furthermore, we analyzed samples from dogs living in the same environment (cohort-2) to compare the nasal and oral microbiota of dogs with different detection jobs and to assess the temporal stability of nasal and oral cavity microbiota.

The oral cavity had a higher bacterial diversity than the nasal cavity. Principal coordinate analysis (PCoA) plots revealed significant differences in microbial communities between the nasal and oral microbiota. In addition, this study was able to show the inter-individual variability in the nasal and oral microbiota of dogs, which can be seen in cohort-1. Though it can be argued that the location of the dogs could attribute to this variability, a similar pattern was also observed in the second cohort of dogs who were all from the same location (cohort-2). As observed in a previous study looking at canine skin microbiota, even the distribution of sequences from chloroplasts though removed from the final analysis, were not uniform between the dogs [26]. The high degree of inter-individual variability in the canine nasal and oral microbiota seen in this study relates to the “personal microbiome” concept suggested in previous studies [26–28].

We also examined the potential role of geographical location on the canine nasal and oral microbiota. Our results suggest that the nasal and oral microbiota can differ based on location. To remove the effect of breed as a potential confounding factor, we analyzed only Labradors from two locations, Alabama and California. Alpha diversity was significantly different based on location for the nasal samples but was not different for the oral samples. Beta diversity was significantly different based on the location in nasal and oral samples from Labradors. Previous studies show that when compared to the gut microbiota, host skin microbiota is more dependent on geographic location [15, 29]. Hence, more detailed and controlled studies are needed to understand if an altered microbiota due to a change in geographical location could bring about changes in physiological functions of the host.

Previous studies had detected Proteobacteria and Firmicutes as the predominant phyla inhabiting the canine nasal cavity [15, 30]. The main phyla in canine nasal cavity in this study were Proteobacteria and Bacteroidetes. Interestingly, there was a large variation based on the individual, with some dogs having Proteobacteria as the abundant phylum and some with Bacteroidetes. Other phyla detected in the canine nasal cavity were Firmicutes, Tenericutes, and GN02 at a lower abundance. The main bacterial classes associated with nasal samples was Gammaproteobacteria, Flavobacteria, and Bacteroidia. A culture-independent study [15] reported the same observation in canine nostrils. Tress et al., reported that Moraxellaceae is abundant in the nasal microbiota of healthy dogs, dogs with nasal neoplasia, and dogs with chronic lymphoplasmacytic or neutrophilic rhinitis [30]. The most frequently observed genera in the nasal cavity of healthy dogs in this study were *Moraxella*, *Mycoplasma*, *Prevotella*, *Helcoccus*, *Cardiobacterium* and unclassified genera within the phylum BD1-5 and the family [Weeksellaceae] respectively. BD1-5 is a candidate phylum which has been reported to have a small genome and is dependent on other members of the community for nutrients. It is a phylum that has not been isolated by culture successfully [31] and it has been previously reported in the canine nasal cavity [30].

In the current study, the predominant phyla found in the canine oral microbiota were Bacteroidetes, Proteobacteria, Firmicutes, and Fusobacteria. This is in concurrence with a previous study by Sturgeon et al., which describes the canine oral microbiota of 6 healthy pet dogs of various breeds based on culture-independent methods [17]. *Porphyromonas* was the predominant genus in oral samples in our study, which is in agreement with previous literature using culture-independent methods [16–18]. In most culture-based studies, *Porphyromonas* has been reported to be of low abundance [19, 32], as noted in a previous study [17], this could be due to the fastidious growth requirements of this organism. Some of the taxa found in the nasal and oral cavity of dogs in this study have been associated with mostly soil or plant samples previously [33, 34]. It is most likely that presence of these taxa is due to the interactions of the host with the outdoor environment.

There are no previous studies detailing the nasal and oral microbiota of working dogs or dogs trained for tasks that require special olfactory skills. In addition, there are no studies examining the variation of canine nasal and oral microbiota over time. The dogs in the cohort-2 were all from a single training facility and were on the same diet. This study did not reveal any significant differences in alpha diversity based on the detection category of dogs. However, there was a significant difference in beta diversity in oral samples based on the type of detection job. LEfSe was able to identify a few bacterial groups that were different between dogs belonging to the three different detection categories. *Cardiobacterium* and *Riemerella* were amongst the taxa that were enriched in the nasal samples from P-NDD and VWD-E dogs. These taxa have been reported as members of the healthy canine nasal microbiota [30, 35]. The genus *Capnocytophaga* was found to be enriched in the oral cavity of P-NDD group of dogs, a member of this genus has been associated with bite wound infections [36]. Similarly, *Aggregatibacter* and *Gemella* were found to be abundant in the oral cavity of SP-E dogs and were detected in healthy canine oral microbiota [37].

Even though we were able to look at only one additional time point approximately 7 weeks from the first sampling, the results suggest that in detection canines, the nasal microbiota may be variable while the oral microbiota is less so. The dogs have been in this facility for at least 6 months; however, most of these dogs were at different locations (Afghanistan, Europe, Iraq, Mexico and various locations in the United States) before being acquired by the facility. The authors could only propose at this point that this could be a driver for the variation, but further studies are needed to validate this.

This study had limitations. A variety of factors including diet, sex, breed, age, and the geographical location could affect the nasal and oral microbiota of dogs. We tried to examine the effect of location in cohort-1 by excluding the effects of confounding factors by focusing on dogs, which were of the same breed. Unfortunately, breed comparisons were not possible in the cohort-1, as the number of animals/breed in each location were very low to make meaningful statistical comparisons. We could not explore the effect of diet, since diet was linked to the location. Most individuals from the same location were on the same diet. Similarly, the effect of age was also difficult to conclude since it was also linked with location. In the second cohort of detection dogs, which were all from the same location and on the same diet, there was a difference in the oral microbiota based on the age and breed of the dogs. Since samples from cohort-1 and cohort-2 were sequenced on different runs; we did not merge the datasets together and introduce a sequencing run bias. Another limitation of the study was that there were fewer animals in each group to perform statistical comparisons and make stronger conclusions from the results. An interesting aspect would be to assess the differences if any in the nasal and oral microbiota of detection dogs to pet dogs. Further studies are needed to know if there are specific groups that are influencing olfactory detection skills in working dogs.

## Conclusion

In this study, we report further characterization of the nasal and oral microbiota of dogs from different geographic locations using next generation sequencing. The results show that the nasal and oral microbiota is distinct and unique in bacterial diversity and community structure. Species richness, bacterial communities, and specific taxa were significantly different between nasal and oral samples. Our results suggest that geographical location may influence the nasal and oral cavity microbiota. This study is also the first study to analyze the nasal and oral microbiota of dogs with specific detection jobs. Initial data shows that there is no significant difference in diversity based on olfactory skills in canine nasal and oral cavity. However, a few distinct bacterial groups were identified using the LEfSe algorithm to be associated with the detection job categories. Our initial data suggest that nasal microbiota may be temporally more variable than oral microbiota in detection canines. Further studies are required to understand the role of the nasal and oral microbiota in canine olfaction.

## Supporting information

S1 Table Metadata of study subjects in cohort 1. (XLSX)

S2 Table Metadata of study subjects in cohort 2. (XLSX)

S3 Table Relative percentages of the most abundant bacterial groups on the various phylogenetic levels (phylum, class, order, family, genus). (XLSX)

S4 Table Relative percentages of the most abundant bacterial groups in the nasal samples at the various phylogenetic levels (phylum, class, order, family, genus) in each detection job category. (XLSX)

S5 Table Relative percentages of the most abundant bacterial groups in the oral samples at the various phylogenetic levels (phylum, class, order, family, genus) in each detection job category. (XLSX)

S6 Table ANOSIM analyses on available metavariables for detection dogs (cohort 2). (XLSX)

S1 Fig Bacterial diversity measures for samples from cohort-2 (sampling time point 1). (A) Alpha diversity: rarefaction analysis (number of observed species) of 16S rRNA gene sequences. Lines represent the mean of each group, while the error bars represent the standard deviations. (B) Beta diversity: Principal coordinate analysis (PCoA) of unweighted UniFrac distances of 16S rRNA genes. Analysis of similarity (ANOSIM) revealed clustering between nasal and oral samples (R = 0.58; *P* = 0.01).
(TIF)

S2 Fig Bacterial phyla in A) nasal and B) oral samples. Most common bacterial phyla identified in nasal and oral samples from healthy dogs sorted by the phylum Bacteroidetes.
(TIF)

## Abbreviations

ANOSIM: analysis of similarity
FDR: false discovery rate
LDA: linear discriminant analysis
LEfSe: linear discriminant analysis effect size
OTU: operational taxonomic units
PCoA: principal coordinates analysis
P-NDD: patrol and narcotics detection dogs
SP-E: explosive detection dogs
VWD-E: vapor wake dogs
