# Supplementary figures and images for: Characterization of the nasal and oral microbiota of detection dogs
Source: PLoS One. 2017 Sep 21;12(9):e0184899. doi: 10.1371/journal.pone.0184899 (PMC5608223; doi:10.1371/journal.pone.0184899)

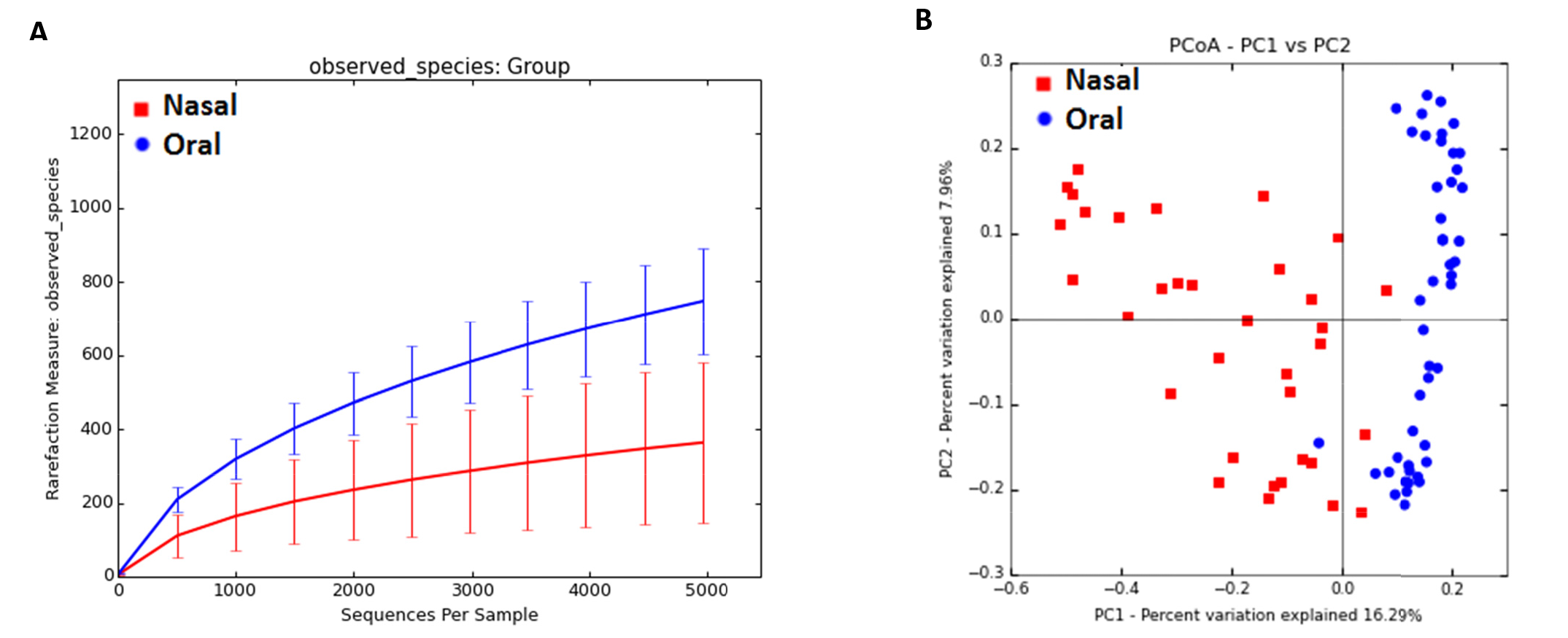

Supplement: S1 Fig — (A) Alpha diversity: rarefaction analysis (number of observed species) of 16S rRNA gene sequences. Lines represent the mean of each group, while the error bars represent the standard deviations. (B) Beta diversity: Principal coordinate analysis (PCoA) of unweighted UniFrac distances of 16S rRNA genes. Analysis of similarity (ANOSIM) revealed clustering between nasal and oral samples (R = 0.58; P = 0.01). (TIF) [file pone.0184899.s007.tif]

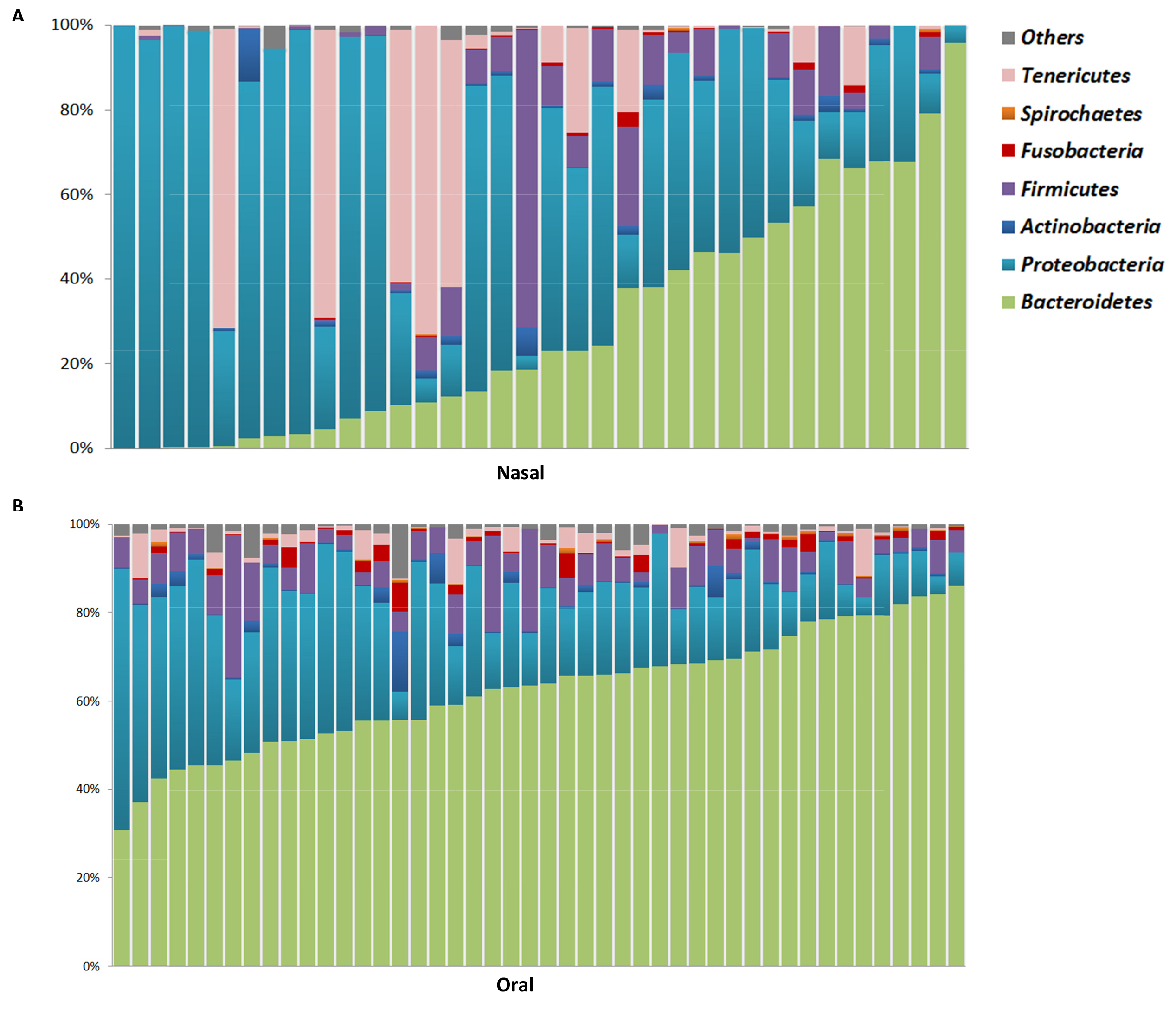

Supplement: S2 Fig — Most common bacterial phyla identified in nasal and oral samples from healthy dogs sorted by the phylum Bacteroidetes. (TIF) [file pone.0184899.s008.tif]
